# Comprehensive analysis, immune, and cordycepin regulation for SOX9 expression in pan-cancers and the matched healthy tissues

**DOI:** 10.3389/fimmu.2023.1149986

**Published:** 2023-03-20

**Authors:** Shuguang Liu, Lisha Yang, Jiewen Fu, Ting Li, Baixu Zhou, Kai Wang, Chunli Wei, Junjiang Fu

**Affiliations:** ^1^ Key Laboratory of Epigenetics and Oncology, Research Center for Preclinical Medicine, Southwest Medical University, Luzhou, China; ^2^ Department of Obstetrics, The Affiliated Hospital of Southwest Medical University, Luzhou, China; ^3^ Department of Gynecology and Obstetrics, Guangdong Women and Children Hospital, Guangzhou, China

**Keywords:** The SOX9 gene, pan-cancers, cordycepin (CD), immune, regulation, drug development

## Abstract

SRY-box transcription factor 9 (SOX9) (OMIM 608160) is a transcription factor. The expression of SOX9 in pan-cancers and the regulation by small molecules in cancer cell lines are unclear. In the current study, we comprehensively analyzed the expression of SOX9 in normal tissues, tumor tissues and their matched healthy tissues in pan-cancers. The study examined the correlation between immunomodulators and immune cell infiltrations in normal and tumor tissues. Cordycepin (CD), an adenosine analog for SOX9 expression regulation, was also conducted on cancer cells. The results found that SOX9 protein is expressed in a variety of organs, including high expression in 13 organs and no expression in only two organs; in 44 tissues, there was high expression in 31 tissues, medium expression in four tissues, low expression in two tissues, and no expression in the other seven tissues. In pan-cancers with 33 cancer types, SOX9 expression was significantly increased in fifteen cancers, including CESC, COAD, ESCA, GBM, KIRP, LGG, LIHC, LUSC, OV, PAAD, READ, STAD, THYM, UCES, and UCS, but significantly decreased in only two cancers (SKCM and TGCT) compared with the matched healthy tissues. It suggests that SOX9 expression is upregulated in the most cancer types (15/33) as a proto-oncogene. The fact that the decrease of SOX9 expression in SKCM and the increase of SOX9 in the cell lines of melanoma inhibit tumorigenicity in both mouse and human *ex vivo* models demonstrates that SOX9 could also be a tumor suppressor. Further analyzing the prognostic values for SOX9 expression in cancer individuals revealed that OS is long in ACC and short in LGG, CESC, and THYM, suggesting that high SOX9 expression is positively correlated with the worst OS in LGG, CESC, and THYM, which could be used as a prognostic maker. In addition, CD inhibited both protein and mRNA expressions of SOX9 in a dose-dependent manner in 22RV1, PC3, and H1975 cells, indicating CD’s anticancer roles likely *via* SOX9 inhibition. Moreover, SOX9 might play an important role in tumor genesis and development by participating in immune infiltration. Altogether, SOX9 could be a biomarker for diagnostics and prognostics for pan-cancers and an emerging target for the development of anticancer drugs.

## 1 Introduction

SRY-box transcription factor 9 (SOX9) (OMIM 608160) is a transcription factor gene that maps to 17q24.3 and encodes 509 amino acids with a molecular mass of 56,137 Da (1). The SOX9 protein as a transcription factor recognizes the CCTTGAG motif along with other HMG-box class DNA-binding protein members, such as SRY (Sex-Determining Region Y) (2). SOX9 is involved in various developmental pathways, including differentiation and progenitor cell development (3, 4). During chondrocyte differentiation, SOX9 acts together with steroidogenic factor 1 to regulate the transcriptional expression of the anti-Muellerian hormone (AMH) gene. Mutations or defects with SOX9 are associated with skeletal malformation syndrome (campomelic dysplasia; OMIM 57 114290) or sex reversal (46,XY Sex Reversal 10; OMIM 57 616425) disorders (5, 6). Campomelic dysplasia is a severe form of autosomal dominant skeletal dysplasia with congenital short and curved long tubular bones. 46,XY Sex Reversal is an XY karyotype in which patients are born looking like normal females but fail to develop secondary sexual characteristics during puberty and have no menstruation.

Subsequently, the role of SOX9 in cancer growth and invasion was revealed. Wang et al. (7) first showed that overexpression of SOX9 promoted tumor growth in xenograft experiments using prostate cancer cells, whereas SOX9 knockdown repressed tumor growth (7). They also found that SOX9 expression was restricted to the basal epithelium of the adult prostate, which begins to be expressed at 19 weeks of gestation, ultimately concluding that SOX9 may allow prostate epithelial cells to grow toward the mesenchyme and then provide basal cellular support for the development and maintenance of ductal epithelial cells. However, SOX9 expression was weak or negative in melanoma specimens but positive in normal skin, and upregulation of SOX9 expression significantly inhibited tumorigenesis in both melanoma-bearing mice and human melanoma *ex vivo* models (8). In melanoma cell lines, treatment with PGD2 (176803) increased SOX9 expression and restored retinoic acid sensitivity. As a proto-oncogene or tumor suppressor gene, SOX9 can induce epithelial–mesenchymal transition (EMT) by regulating the tumor microenvironment (TME) to acquire stem cell characteristics, which are dependent on cancer type (9–11). Thus, activation of the SOX9 pathway may play crucial roles in cancer development and progression (10). Over the past decade, SOX9 has been intensively studied in the field of cancer.

Besides, SOX9 has been shown to be closely associated with tumor immunity. Yuan et al. found that SOX9 expression in thymoma was negatively correlated with target genes related to Th17 cell differentiation, primary immunodeficiency, PD-L1 expression, and T-cell receptor signaling pathways, suggesting that SOX9 may be associated with immune dysregulation in thymoma (12). In the progression of breast cancer, SOX9 triggers tumorigenesis by facilitating the immune escape of tumor cells (13). Ashkenazi et al. indicated that the downregulation of SOX9 contributed to reduced T-cell cytotoxicity (14). In our opinion, the immunopromotive and immunosuppressive effects of SOX9 on tumors may be attributed to the degree to which different tumor types act on the tumor microenvironment.

Cordycepin (CD) is an adenosine analog isolated from the traditional Chinese medicine cordyceps sinensis with a wide range of biological activities, including anti-inflammatory (15), anti-tumor (16), immunomodulatory (17), etc. In our previous studies, it was shown that CD downregulated transcription factors to inhibit the migration and invasion of triple-negative breast cancer cells as well as the progression of drug-resistant non-small cell lung cancer by regulating the AMPK signaling pathway (18, 19). In addition, we found that CD was also able to remarkably reduce the syncytium formation and fluorescence intensity of the SARS-CoV-2 spike pseudotyped virus that invaded 293-ACE2 cells, indicating its anti-COVID potential (20, 21). However, the expression and immunomodulation of SOX9 in pan-cancer and the regulation of the small-molecule drug CD in cancer cell lines are not clear.

In the current study, we thoroughly analyzed SOX9 expression in normal and tumor tissues, matched healthy tissues, and performed correlation analysis with immunomodulators and immune cell infiltration in pan-cancer. The regulation of SOX9 expression by the adenosine analog CD has also been studied in cancer cells, including prostate cancer cell lines.

## 2 Materials and methods

### 2.1 Online data collection

**Figure 1:**
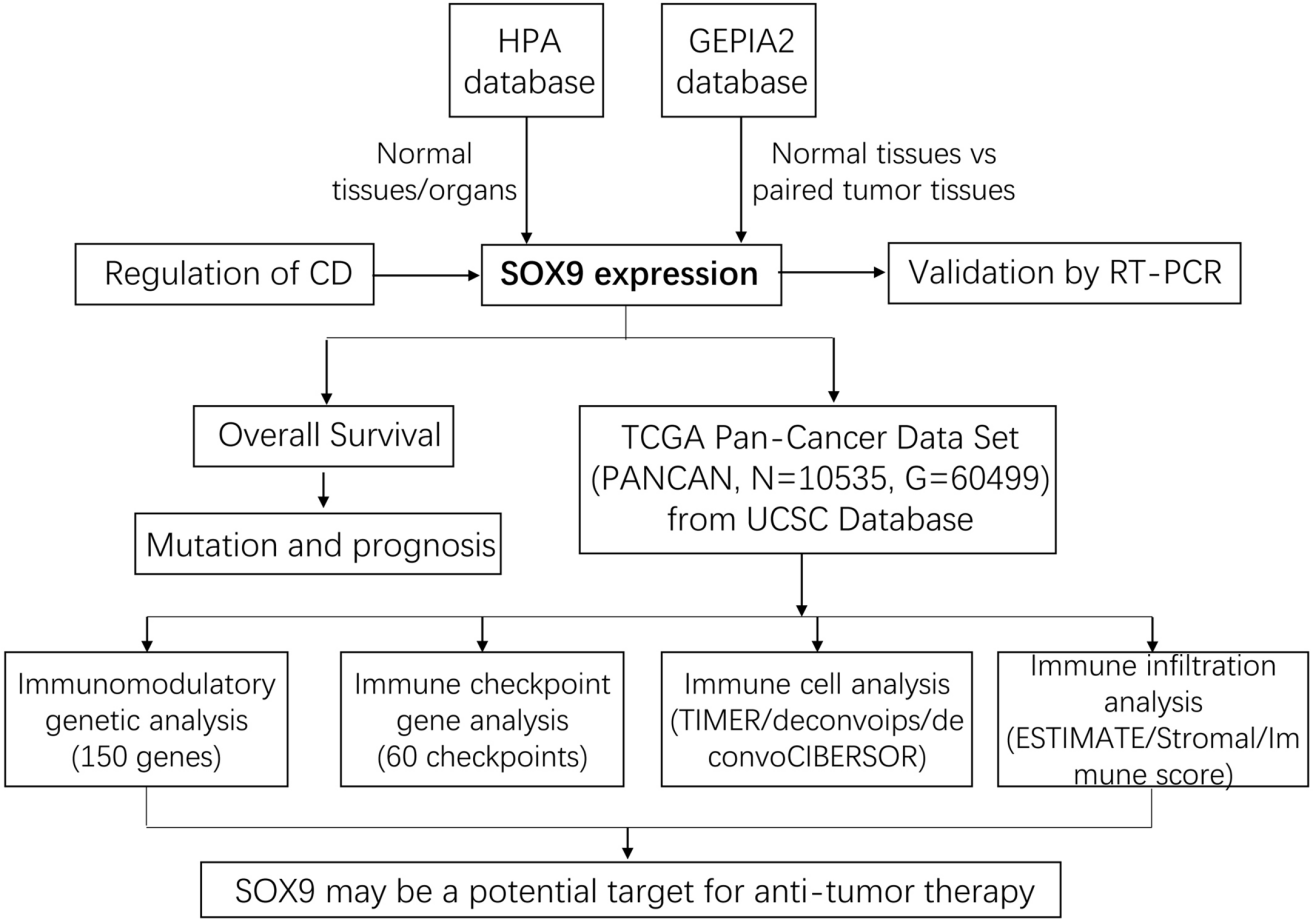
The workflow of our study. First, SOX9 expression in normal tissues and pan-cancer was analyzed using the HPA and GEPIA2 databases, which was further validated by RT-PCR. Subsequently, the overall survival analysis and mutation and prognosis analysis of tumor patients with SOX9 were performed comprehensively. The regulation of SOX9 by a small molecule compound, cordycepin **(CD)**, was explored. Finally, pan-cancer data were collected again from the UCSC database for immunomodulatory gene analysis, immune checkpoint gene analysis, immune cell analysis, and immune infiltration analysis, respectively.

The Human Protein Atlas (HPA) database (https://www.proteinatlas.org/Ensembl ID: ENSG00000125398) was applied to search for mRNA and protein expression of SOX9 in normal tissues. The immunohistochemical and immunofluorescence images of SOX9 in normal and tumor tissues were downloaded from HPA, too (22, 23). Gene expression profiles were obtained from the online Gene Expression Profile Interaction Analysis (GEPIA 2 dataset; http://gepia2.cancer-pku.cn/#index) (24–26) and were employed to compare SOX9 expression in tumors and corresponding healthy tissues. Mutational hot spot analysis of SOX9 as well as survival analysis were used in cBioPortal (27). Additionally, we downloaded the pan-cancer dataset from the UCSC (https://xenabrowser.net/) database: TCGA Pan-Cancer (PANCAN, N = 10,535; G = 60,499). The workflow of our study is shown in **Figure 1**.

### 2.2 HPA analysis

SOX9 mRNA and its protein expression in healthy and tumor tissues from HPA (https://www.proteinatlas.org/) were analyzed (23). *SOX9* mRNA expression levels in healthy tissues were found in HPA, GTEx, and FANTOM5, while normalized expression in tissues and distinct blood cells was obtained from the three databases mentioned above (v20.proteinatlas.org/about/assays+annotation#normalization_rna).

### 2.3 GEPIA and prognostic analysis of SOX9

*SOX9* mRNA expression in 5,540 healthy and 9,663 tumor tissues and the relationship between SOX9 expression levels and median overall survival (OS) were analyzed by GEPIA (25). A correlation analysis of SOX9 expression and immune regulation was performed. Data for pan-cancer (PANCAN, N = 10,535; G = 60499) was downloaded from the UCSC database (http://xenabrowser.net/).

### 2.4 Cell culture and small molecular compound cordycepin treatment

Prostate cancer cells PC3 and 22RV1 and lung cancer cell H1975 were obtained from the Cell Bank of the Research Center for Preclinical Medicine, Southwest Medical University, and these cells were purchased from ATCC, USA. H1975 and PC3 cells were cultured in RPMI 1640 medium (Gibico, USA) containing 10% fetal bovine serum (FBS) and 1% penicillin/streptomycin. 22RV1 cells were cultured in DMEM medium (Gibico, USA), which contains 15% fetal bovine serum (FBS) and 1% penicillin/streptomycin. All cells were cultured in a 37°C incubator with 5% CO_2_. CD was obtained from Chengdu Must Bio-Technology Co. Ltd. (Chengdu, Sichuan, China), which has been used previously (18, 28, 29). Cells were inoculated in 12-well plates and treated with CD at final concentrations of 0, 10, 20, and 40 µM for 24 h. Protein was collected, and expression levels were monitored by Western blot. Total RNA was extracted by reverse transcription (29, 30).

### 2.5 Western blot assays

Cells were lysed in EBC buffer and 2×SDS loading buffer to collect proteins. The protein samples were boiled at 100°C for 5 min and then electrophoresed in the Bio-Rad Mini PROTEAN Tetra System (Bio-Rad, USA). After electrophoresis, the proteins were transferred to the PVDF membrane under ice bath conditions, and then the membrane was washed twice with 1×TBST. The membrane was blocked with 5% free-fat milk for 2 h at room temperature. The primary antibodies to SOX9 (67439-1-Ig, Proteintech) and HSP90 (ab203126, Abcam) were diluted with 2% free-fat milk at ratios of 1:4,000 and 1: 10,000, respectively, and then incubated overnight at 4°C. Membrane was washed thrice for 15 min and incubated the secondary antibodies for 2 h at room temperature. After another three times washing, the bands were solarized and imaged using the Syngene G: BOX Imaging System (Cambridge, UK) (19, 31).

### 2.6 RT-PCR analysis

The total RNA was extracted using a TIANGEN kit (cat. no.: #DP419, TIANGEN, China), then reversely transcribed into cDNA using a reverse transcription kit (TOYOBO, China). The forward primer 5’-gaggaagtcggtgaagaacg-3’ and the reverse primer 5’-atcgaaggtctcgatgttgg-3’ for SOX9 were designed on the Primer3 online primer design website. The product size for SOX9 is 337 bp. ACTB was used as an internal control. PCR amplification was conducted using a Veriti 96-well thermal cycler (ABI, USA); it is worth noting that the amplification number for SOX9 did not exceed 30 cycles. After PCR reactions were completed, agarose electrophoresis for the amplified products was performed on 1.5% agarose gel (30).

For the LUSC samples’ quantitative RT-PCR, the tumor samples and the matched healthy tissue samples were collected from Chinese individuals (seven pairs of samples) and the RT-PCR analysis was performed as mentioned above. This study was approved by the Ethical Committee of Southwest Medical University.

### 2.7 Immunomodulatory genetic analysis

The expression data of the *SOX9* gene and 150 marker genes of five immune pathways (chemokines (41), receptors (18), MHCs (21), immunoinhibitors (24) and immunostimulators (46)) in each tumor sample were extracted from the downloaded pan-cancer dataset (TCGA Pan-Cancer (PANCAN, N = 10,535; G = 60,499)), filtered all normal samples, and a log2(x + 0.001) transformation was performed for each expression value. Finally, a Pearson correlation was calculated between SOX9 and the five types of marker genes.

### 2.8 Immune checkpoint gene analysis

The expression data of the *SOX9* gene and 60 marker genes of two types of immune checkpoint pathway genes (inhibitory (24), stimulatory (36)) in pan-cancer were extracted from the downloaded pan-cancer dataset (TCGA Pan-Cancer (PANCAN, N = 10,535; G = 60,499)), and all normal samples were filtered. A log2(x + 0.001) transformation was performed for each expression value, and finally the Pearson correlation of SOX9 with marker genes of five types of immune pathways was calculated.

### 2.9 Immunocytometric analysis

Expression data of the *SOX9* gene in each sample were extracted from the downloaded pan-cancer dataset (TCGA Pan-Cancer (PANCAN, N = 10,535; G = 60,499)) and a log2(x + 0.001) transformation was performed for each expression value. The expression profile was mapped to GeneSymbol and reassessed separately using the R package IOBR (version 0.99.9) of the TIMER, deconvo_ips, and deconvo_CIBERSOR methods to reassess the immune cell infiltration score of each tumor in each patient based on gene expression.

### 2.10 Immune infiltration analysis

The expression data of the *SOX9* gene in each sample were extracted from the downloaded pan-cancer dataset (PANCAN, N = 10,535; G = 60,499); and a log2(x + 0.001) transformation was performed for each expression value, from which the gene expression profile of each tumor was extracted separately and the expression profile was mapped to GeneSymbol. Stromal, immune, and ESTIMATE scores were calculated for each tumor in each patient using the R package ESTIMATE (version 1.0.13).

### 2.11 Statistical analysis

The SOX9 expression levels of all individuals in the survival analysis were separated into high and low expression groups using the median expression of overall survival (OS). Logrank with P <0.05 was considered a significant difference.

## 3 Results

### 3.1 SOX9 expression in human organs

**Figure 2:**
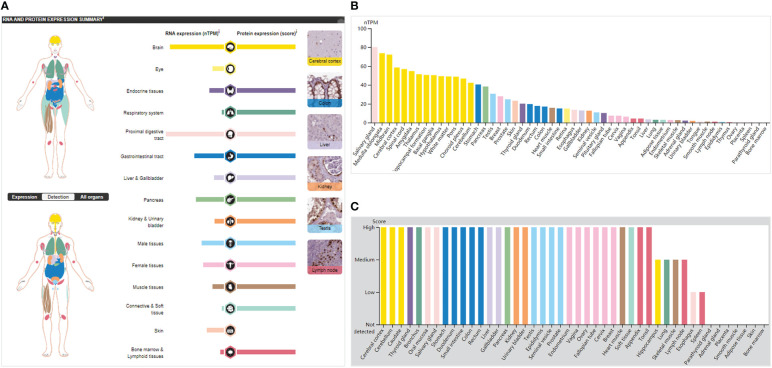
SOX9 expression in normal tissues/organs. **(A)** The general situation of SOX9 mRNA and protein expression. Color-coding lists are based on different tissue groups, and each group comprises tissues with similar functional characteristics. The image on the right shows the immunohistochemical (IHC) staining values of SOX9 in normal tissues. **(B)** mRNA expression of SOX9 in normal tissues, indicated by nTPM (normalized transcripts per million). **(C)** SOX9 protein expression levels in normal tissues by IHC score.

*SOX9* mRNA was expressed non-specifically in many human tissues. For example, it was highly expressed in the proximal digestive tract (salivary glands) and brain, moderately expressed in the gastrointestinal tract (stomach), pancreas, male tissues (prostate and testis), female tissues (breast), and skin, but lowly expressed in tissues such as the kidney and gallbladder (**Figures 2A,B**). The SOX9 protein was highly expressed in 13 organs and not expressed in only two organs (eye and skin) (**Figure 2A**); it was highly expressed in 31 tissues, expressed in four tissues, lowly expressed in two tissues, and not expressed in the other seven tissues (**Figures 2A, C**). This broad protein expression suggests an important role for SOX9 in multiple tissues/organs.

### 3.2 SOX9 expression in pan-cancers and the matched healthy tissues

**Figure 3:**
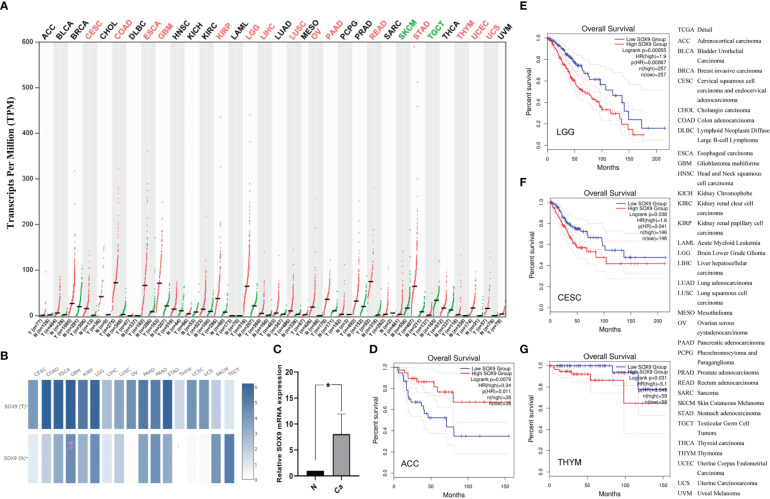
Expressions and prognostic values of SOX9 expression in pan-cancers and the matched healthy tissues. **(A)** The profiles of SOX9 in 33 types of cancer with dot plots. **(B)** The profiles of SOX9 with significant changes in 17 cancer types using heatmaps. “T” indicates tumors, and “N” indicates the matched healthy tissues. **(C)** Verification of LUSC samples by quantitative RT-PCR. *P <0.01. The prognostic values of SOX9 expressions in ACC **(D)**, LGG **(E)**, CESC **(F)**, and THYM **(G)**. The right panel provides a full description of all cancer types.

In 33 cancer types, SOX9 expression was a significant increase in COAD (colon adenocarcinoma), CESC (cervical squamous cell carcinoma and endocervical adenocarcinoma), ESCA (esophageal carcinoma), GBM (glioblastoma multiforme), KIRP (kidney renal papillary cell carcinoma), LIHC (liver hepatocellular carcinoma), LGG (brain lower grade glioma), LUSC (lung squamous cell carcinoma), OV (ovarian serous cystadenocarcinoma), PAAD (pancreatic adenocarcinoma), READ (prostate adenocarcinoma), STAD (stomach adenocarcinoma), THYM (thymoma), UCES (uterine corpus endometrial carcinoma), and UCS (uterine carcinosarcoma), but significant decrease only in SKCM (skin cutaneous melanoma) and TGCT (testicular germ cell tumors) compared with the matched healthy tissues (**Figures 3A, B**). Higher expression of the *SOX9* gene in the LUSC tumor tissues was verified when compared with the matched normal tissues (**Figure 3C**). Thus, SOX9 expression was upregulated in most cancers.

### 3.3 Prognostic values for SOX9 expression in pan-cancer

Further analysis of the prognostic value of SOX9 expression in individuals with cancer revealed that overall survival was longer in ACC (**Figure 3D**) (adrenocortical carcinoma) and shorter in LGG (**Figure 3E**), CESC (**Figure 3F**), and THYM (**Figure 3G**) when SOX9 was highly expressed in pan-cancer compared with the matched healthy tissues. Consequently, the high expression of SOX9 was positively correlated with the poor prognosis of LGG, CESC, and THYM, which may be a prognostic factor.

### 3.4 SOX9 mutations and their prognostics

**Figure 4:**
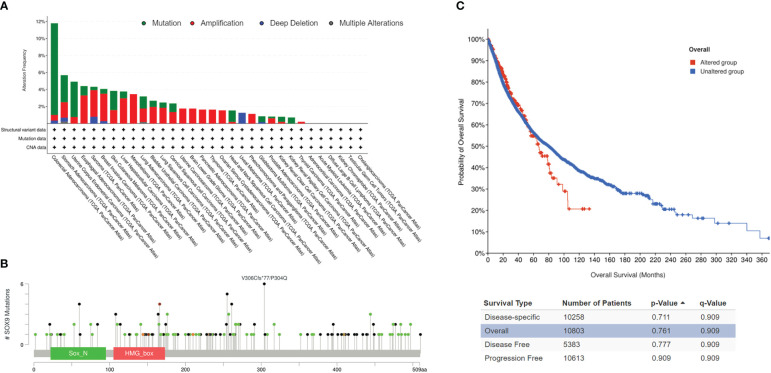
SOX9 mutations in pan-cancers. **(A)** Overview of SOX9 mutations in pan-cancers. Different colors indicate different types of mutations. **(B)** SOX9 mutations and locations in pan-cancers. **(C)** Correlation of survivals between the SOX9 mutated group (red) and unaltered group (blue) in pan-cancers.

cBioPortal analysis in 26 cancer types revealed that SOX9 mutations are highest in COAD with 11.78%, including mutations at 10.77% in 64 cases, amplification at 0.67% in four cases, and deep deletion at 0.34% in two cases, and lowest in THCA (thyroid carcinoma) with 0.2% (amplification of 0.2% in one case) (**Figure 4A**). No SOX9 mutation was found in the other six cancer types, including ACC, KICH (kidney chromophobe), LAML (acute myeloid leukemia), DLBC (diffuse large B-cell lymphoma), CHOL (cholangiocarcinoma), and TGCT (**Figure 4A**). A total of 170 mutations (somatic mutation frequency: 1.4%) were found, including 89 missenses, 69 truncations, nine inframes, and three splices along the whole *SOX9* gene (**Figure 4B**).

Survivals for disease-specific, overall, disease-free, and progression-free conditions revealed no significant difference in the mutated group compared with the unaltered group of SOX9, although median months were much shorter (**Figure 4C**, p >0.05). These data suggested that SOX9 was mutated in most cancers but did not have prognostic significance.

### 3.5 Treatment with CD inhibits SOX9 expression in both protein and mRNA in different cancer cells

**Figure 5:**
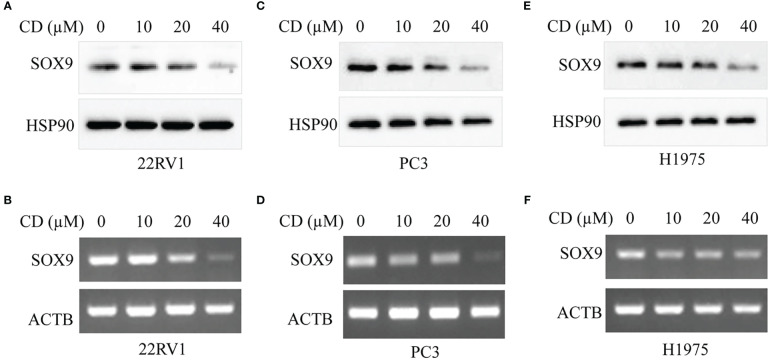
CD inhibits the expression of SOX9 in different tumor cells. **(A)** Protein expression levels of SOX9 in prostate cancer cell 22RV1 after CD treatment. **(B)** mRNA expression level of SOX9 in prostate cancer cell 22RV1 after CD treatment. **(C)** Protein expression levels of SOX9 in prostate cancer cell PC3 after CD treatment. **(D)** mRNA expression level of SOX9 in prostate cancer cell PC3 after CD treatment. **(E)** Protein expression level of SOX9 in lung cancer cell H1975 after CD treatment. **(F)** mRNA expression level of SOX9 in lung cancer cell H1975 after CD treatment.

We then analyzed the effect of CD on SOX9 expression levels in tumor cells and showed that CD dose-dependently decreased the protein of SOX9 and its mRNA expression levels in 22RV1 (**Figures 5A, B**), PC3 (**Figures 5C, D**), and H1975 (**Figures 5E, F**) cells, indicating that CD inhibited SOX9 expression in tumor cells, especially in prostate cancer cells.

### 3.6 SOX9 expression is associated with immune cell infiltration in pan-cancer

**Figure 6:**
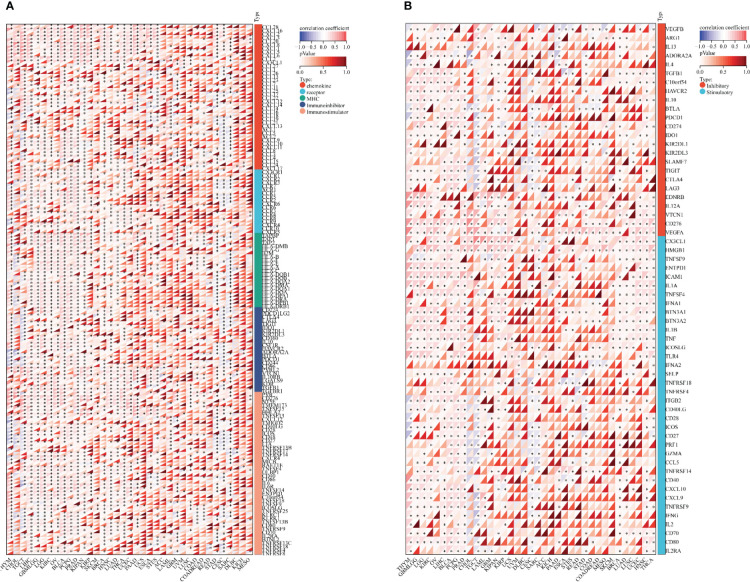
Bioinformatics analysis of the immunoregulatory actions of SOX9 in several cancer types. **(A)** Correlation between SOX9 and 150 genes of five classes of immune pathways (41 chemokines, 18 receptors, 21 MHCs, 24 immunoinhibitors, and 46 immunostimulators). *P <0.05. **(B)** Correlation between SOX9 and 60 genes of two types of immune checkpoint pathways.

We first collected the *SOX9* gene and 60 genes of two immune checkpoint pathways and 150 genes of five immune pathways for analysis of immunoregulation genes, immune checkpoints, immunocytes, and immune infiltration. In the analysis, we detected that SOX9 expression had a positive association with lots of immune regulatory genes, including ADORA2A, TMIGD2, TGFB1, TMEM173, TNFRSF18, IL6R, IL10RB in THYM, CHOL, TGCT, PAAD, ESCA, ACC, LAML, and CESC (**Figure 6A**; **Supplementary Table 1**). In addition, SOX9 expression was reciprocally exclusive with several tumor immune checkpoints, such as CD27, CTLA4, LAG3, TIGIT, IL10, CSF1R, ADORA2A, CD244, etc. (**Figure 6B**; **Supplementary Table 2**).

**Figure 7:**
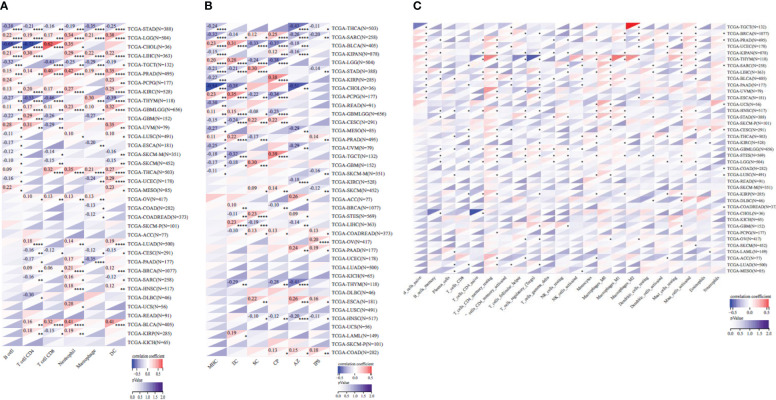
Pearson’s correlation coefficient of the SOX9 expression with tumor-immune systems in several cancer types. **(A)** Correlation between SOX9 and six tumor-interrelated immune cells counted with TIMER. **(B)** Correlation between SOX9 and six tumor-related immune cells counted with deconvo_ips. **(C)** Correlation between SOX9 and 22 tumor-correlative immune cells calculated with the deconvo_CIBERSOR. *P <0.05; **P <0.005; ***P <0.001; ****P <0.0001. The full names of cancer types are shown in **Figure 2**.

Based on *SOX9* gene expression, we reappraised the invasion scores of six immune cells (lymphocyte T CD4, lymphocyte B, macrophage, lymphocyte T CD8, neutrophil, and dendritic cells) for 9,406 tumor samples in 36 cancer types and six immune cells (SC, MHC, EC, IPS, CP, and AZ) and 22 class immunocytes in 9,555 cancer specimens from 39 neoplasm types. Results showed that the SOX9 expression was sensibly related to immune infiltration in 26 tumor species (TCGA-BLCA (N = 405), TCGA-BRCA (N = 1,077), TCGA-CESC (N = 291), TCGA-CHOL (N = 36), TCGA-COAD (N = 282), TCGA-COADREAD (N = 373), TCGA-ESCA (N = 181), TCGA-GBM (N = 152), TCGA-GBMLGG (N = 656), TCGA-HNSC (N = 517), TCGA-KIRC (N = 528), TCGA-KIRP (N = 285), TCGA-LGG (N = 504), TCGA-LIHC (N = 363), TCGA-MESO (N = 85), TCGA-OV (N = 417), TCGA-PAAD (N = 177), TCGA-PCPG (N = 177), TCGA-PRAD (N = 495), TCGA-SARC (N = 258), TCGA-SKCM (N = 452), TCGA-STAD (N = 388), TCGA-TGCT (N = 132), TCGA-THCA (N = 503), TCGA-THYM (N = 118), TCGA-UVM (N = 79)) (**Figures 7A–C**; **Supplementary Tables 3**–**5**).

**Figure 8:**
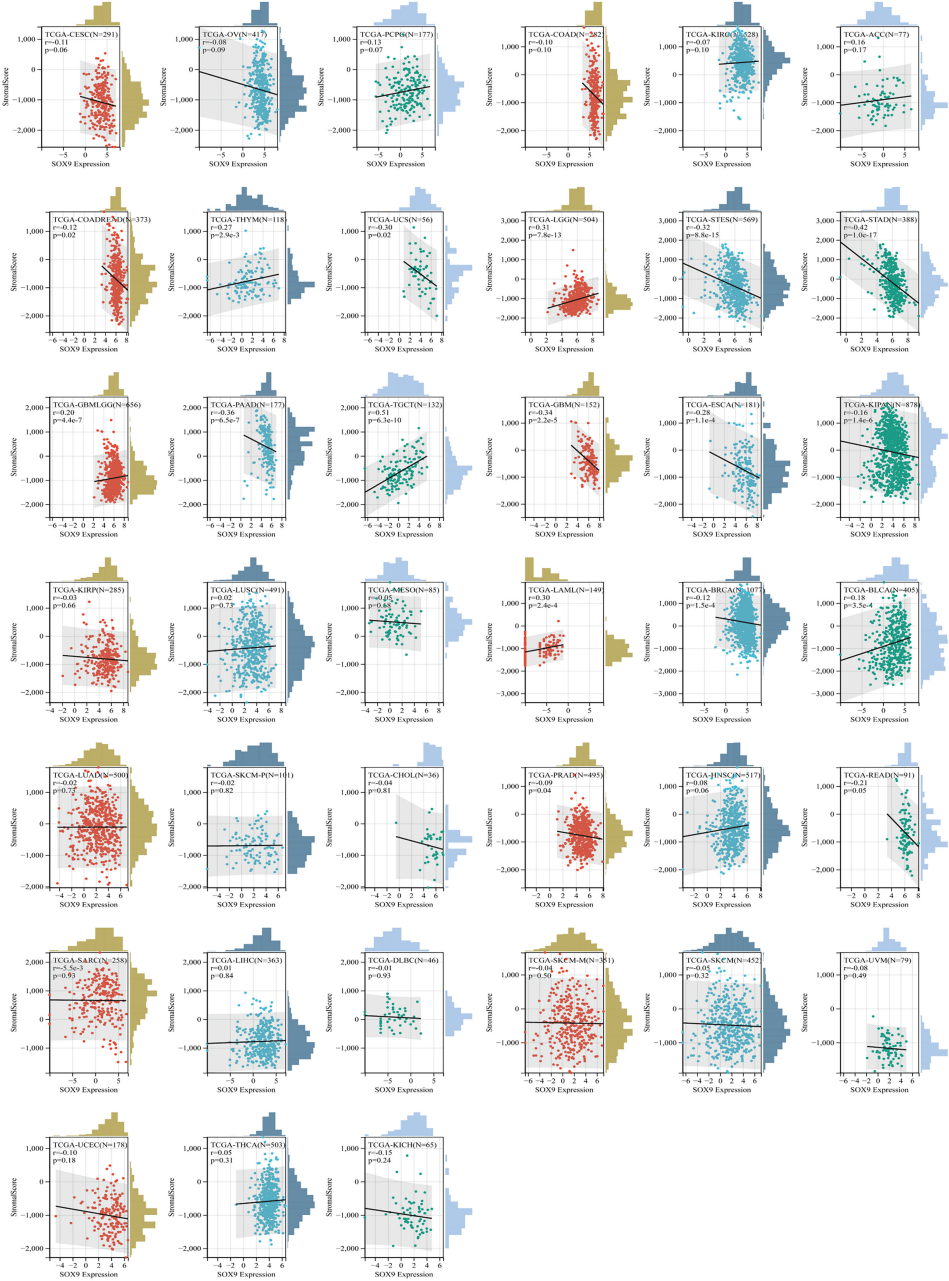
The correlation between SOX9 expression and the immune infiltration score indicated several cancer types.

In addition, we detected the relevance between the state of immune invasion and SOX9 expression in cancer. We discovered that the *SOX9* gene expression was notably interrelated with immune invasion in 17 neoplasm species, indicating six significant positive correlations (TCGA-GBMLGG (N = 656, R = 0.20, P = 4.4e−7), TCGA-LGG (N = 504, R = 0.31, P = 7.8e−13), TCGA-LAML (N = 149, R = 0.30, P = 2.4e−4), TCGA-THYM (N = 118, R = 0.27, P = 2.9e−3), TCGA-TGCT (N = 132, R = 0.51, P = 6.3e−10), TCGA-BLCA (N = 405, R = 0.18, P = 3.5e−4)) and 11 significant negative associations (TCGA-GBM (N = 152, R = −0.34, P = 2.2e−5), TCGA-COADREAD (N = 373, R = −0.12, P = 0.02), TCGA-BRCA (N = 1,077, R = −0.12, P = 1.5e−4), TCGA-ESCA (N = 181, R = −0.28, P = 1.1e−4), TCGA-STES (N = 569, R = −0.32, P = 8.8e−15), TCGA-KIPAN (N = 878, R = −0.16, P = 1.4e−6), TCGA-STAD (N = 388, R = −0.42, P = 1.0e−17), TCGA-PRAD (N = 495, R = −0.09, P = 0.04), TCGA-READ (N = 91, R = −0.21, P = 0.05), TCGA-PAAD (N = 177, R = −0.36, P = 6.5e−7), TCGA-UCS (N = 56, R = −0.30, P = 0.02)) by assaying the connection among *SOX9* and immune infiltration marks in 9,555 tumor specimens from 39 cancers (**Figure 8**; **Supplementary Table 6**).

These results suggest that it is probable for SOX9 to be sensibly interrelated with immune infiltration of neoplasms and negatively associated with tumor immunosuppression. We know that tumor-related immune cells infiltrating tumor tissues affect TME and can help tumor cells escape immune surveillance, thus promoting the malignant progression of tumors (32–34). Additionally, our studies indicated that the expression of SOX9 was negatively correlated with multiple immunosuppressants, and many cancer species related to the expression of SOX9 were highly malignant, such as COAD, LAML, ESCA, etc., implying that the correlation between SOX9 expression and immune cell infiltration in pan-cancer may be related to the malignancy of the tumor.

## 4 Discussion

In the current study, we revealed that SOX9 protein was expressed in multiple organs. For example, SOX9 was highly expressed in 13 organs and absent in only two organs (eye and skin); it was highly expressed in 31 of 44 tissues, expressed in four tissues, lowly expressed in two tissues, and absent in the other seven tissues, indicating an important role for SOX9 in multiple tissues/organs. This contrasts with the positive SOX9 expression results in healthy skin reported by Passeron et al. (8). In addition, we did not observe SOX9 protein expression but only saw significant SOX9 mRNA expression (23.3 nTPM), which implies that the IHC score may be inaccurate. We found that the *SOX9* gene was highly expressed in COAD, ESCA, CESC, GBM, KIRP, LGG, LIHC, LUSC, OV, PAAD, READ, STAD, THYM, UCES, and UCS, and lowly expressed in SKCM and TGCT, suggesting that SOX9 may be a pro-oncogene in most cancer types. It has also been reported in the literature that reduced expression of SOX9 in SKCM and overexpression of SOX9 in melanoma cell lines suppressed tumorigenesis in both mouse and human *in vitro* models (8), indicating that SOX9 may be a tumor suppressor gene in both cancer types. Prognostic analysis showed that SOX9 expression was positively correlated with the prognosis of ACC patients and negatively correlated with the prognosis of LGG, CESC, and THYM patients, which suggests that SOX9 is likely to be an oncogene, making it an important factor affecting the prognosis of LGG, CESC, and THYM patients.

The interaction between tumors and immunity is a hot and difficult point that has been studied but has never been deeply clarified (35). Many cancers use embryonic genes to grow wildly and escape the monitoring of the immune system. SOX9 is upregulated in many tumors, as described above in 15 cancers. However, the role of SOX9 in mediating an immunosuppressive tumor microenvironment is still unclear (36, 37). Next, we explored the immunomodulatory role of SOX9 in cancer. Bioinformatics results showed that SOX9 was positively associated with immunomodulatory genes such as ADORA2A, TMIGD2, TGFB1, TMEM173, TNFRSF18, IL6R, IL10RB in THYM, CHOL, TGCT, PAAD, ESCA, ACC, LAML, and CESC, indicating the immune-promoting role of SOX9. Because ADORA2A is an adenosine receptor distributed on the surface of immune cells (NK, CD4+ and CD8+ T cells, and macrophages) (38). In the tumor microenvironment (TME), ADORA2A promotes adenosine signal transduction, inhibits infiltration of CD8+ T cells and NK cells, and promotes tumor progression (39). TMIGD2 is widely expressed in T cells, B cell DCs, and monocytes and has been shown to promote angiogenesis and increase actin filament formation, leading to cell adhesion and inhibition of cell migration (40). PD-L1 is highly expressed in most cancers, and the PD-L1/PD-1 signaling pathway contributes to cancer evasion by T-cell immunity (41). We found that SOX9 negatively correlated with CD8+ T cells, activated NK cells, M2 macrophages, and other tumor-infiltrating immune cells. It is well known that TME is composed of vascular endothelial cells, fibroblasts, and immune cells, which promote oncogenic gene expression and block the immunomodulatory effects of distinct immune cells. Both CD8+ T cells and activated NK cells exhibit strong tumor-killing effects (42), and M2 macrophages play a role in suppressing immune responses in the tumor microenvironment (43). These results suggest that SOX9 expression may be able to regulate TME homeostasis by modulating various immune cells and immunomodulatory genes. The immune checkpoint pathway is a mechanism used by tumor cells to disguise themselves as normal components of the human body (44–46). In addition, SOX9 was mutually exclusive with a variety of tumor immune checkpoints (CD27, CTLA4, LAG3, TIGIT, IL10, CSF1R, ADORA2A, CD244, etc.), further suggesting that SOX9 may be a novel target with great potential in tumor immunotherapy. Thus, SOX9 may play an important role in tumor genesis and development by participating in immune infiltration. Moreover, the correlation between SOX9 expression and tumor immune cell infiltration may be related to the malignancy of the tumor. The bioinformatics approach we used in this study can rapidly predict the role of expected target molecules in disease progression and the potential association between molecules based on a large amount of sequencing data. However, the amount of sample size may also cause inconsistency between the prediction results and experimental results, thus generating errors.

CD is an adenosine analog with wide pharmacological effects and maybe resistance to a variety of tumors (18, 19, 47) and viruses (48–50), including SARS-CoV-2 (20, 29, 51, 52). We analyzed the role of CD in different tumor cells and found that CD concentration-dependently decreased SOX9 protein and mRNA expression in 22RV1, PC3, and H1975, suggesting that the anticancer effect of CD may be associated with SOX9 inhibition. CD has been shown to be an immunomodulator to suppress T-cell activity, reduce IL-2 levels, and to increase IL-10 levels, along with affecting the regulation of immune cells and cytokine networks (53). SOX9’s tumor immunomodulatory role will be further elucidated in future experiments.

## Conclusions

Collectively, SOX9 can be used as a diagnostic and prognostic marker for many types of tumors. Notably, high SOX9 expression in pan-cancer may predict the tumor immunosuppressive microenvironment, suggesting an important role for SOX9 in tumor immune regulation. CD significantly inhibits SOX9 expression in a variety of tumor cells and targeting SOX9 with CD is more promising as a strategy for cancer therapy.

## Data availability statement

The original contributions shown in the study are included in the article. Further inquiries can be directed to the corresponding authors.

## Ethics statement

The study was approved by the Ethical Committee of Southwest Medical University.

## Author contributions

JJF conceived and coordinated the study. SL, LY, JWF, TL, BZ, CW, KW, and JJF conducted experiments, analyzed and interpreted data. SL, KW, and JJF wrote and edited the manuscript. All authors contributed to the article and approved the submitted version.
